# Signaled night awakening and its association with social information processing and socio-emotional development across the first two years

**DOI:** 10.1093/sleep/zsab179

**Published:** 2021-07-16

**Authors:** Tiina E Mäkelä, Anneli Kylliäinen, Outi Saarenpää-Heikkilä, E Juulia Paavonen, Tiina Paunio, Jukka M Leppänen, Mikko J Peltola

**Affiliations:** 1 Psychology, Faculty of Social Sciences, Tampere University, Tampere, Finland; 2 Faculty of Medicine and Life Sciences, Tampere University, Tampere, Finland; 3 Department of Pediatrics, Tampere University Hospital, Tampere, Finland; 4 Public Health and Welfare, Finnish Institute for Health and Welfare, Helsinki, Finland; 5 Pediatric Research Center, Child Psychiatry, University of Helsinki and Helsinki University Hospital, Helsinki, Finland; 6 Psychiatry and SleepWell Research Program, Faculty of Medicine, University of Helsinki and Helsinki University Hospital, Helsinki, Finland; 7 Department of Psychology and Speech-Language Pathology, University of Turku, Turku, Finland

**Keywords:** night awakening, fragmented sleep, social cognition, social information processing, socio-emotional development, infancy

## Abstract

**Study Objectives:**

Night awakening is common in infancy, and some infants continue to have signaled night awakenings throughout early childhood. However, the influence of signaled night awakening on children’s social development is less explored. In the present study, longitudinal associations between signaled night awakening, social information processing, and socio-emotional development were measured within the CHILD-SLEEP birth cohort in two groups formed based on parent-reported night awakenings.

**Methods:**

At 8 months, there were 77 infants in the waking group (≥3 awakenings) and 69 infants in the nonwaking group (≤1 awakening). At 8 and 24 months, social information processing was measured as children’s attention to neutral and emotional faces, and at 24 months, parent-reported socio-emotional behavior was measured with the Brief Infant-Toddler Social and Emotional Assessment (BITSEA) questionnaire.

**Results:**

The two groups showed different patterns of attention to emotional faces. The waking group had a more pronounced attentional bias to fearful versus happy faces, whereas in the nonwaking group, attention to fearful and happy faces did not differ. In addition, at 24 months, the waking group had more dysregulation problems and lower social competence than the nonwaking group, but no clear differences in internalizing or externalizing problems were found.

**Conclusions:**

Our results contribute to the literature by showing that during the first 2 years of life, signaled night awakening is associated with social information processing and socio-emotional behavior.

**Statement of Significance**

Our study focused on associations between signaled night awakening, social information processing, and socio-emotional development in infancy, a topic that has received little attention in previous studies even though signaled night awakening is a common concern for parents and it has a potentially critical influence on children’s development. We studied social information processing longitudinally at the age of 8 and 24 months and parents reported children’s socio-emotional behavior at 24 months. Our study provides a novel contribution regarding the influence of signaled night awakening on children’s social information processing and social development already in infancy by showing that children with signaled night awakenings show a different pattern of attention to emotional faces and they have lower parent-reported social competence and more behavioral dysregulation symptoms.

## Introduction

Sleep is an important part of healthy development and is associated with multiple developmental outcomes in childhood. Sleep in infancy has unique characteristics, including the fragmented nature of infant sleep caused by frequent night awakenings. Short night awakenings between sleep cycles are normative [1] and seem not to be problematic for infants who can soothe themselves back to sleep without parental assistance [2–4]. Night awakenings that are signaled to parents, for example by crying, and which require parental assistance may cause problems for the wellbeing of the whole family [3], and are most often reported by parents when evaluating their infant’s quality of sleep [5]. Night awakenings that require parental assistance are referred to as signaled night awakening. Increased night awakenings have been associated with parent- and child-related factors, such as breastfeeding, co-sleeping with the infant, high parental involvement when putting the child to sleep, and the child’s negative emotionality [6–8]. Prolonged and recurrent signaled night awakenings can be persistent in infants [6, 9, 10] and even in children aged 4–6 years [5, 11]. In addition, it has been proposed that signaled night awakening could be a precursor for later appearing sleep difficulties [2] and a risk factor for the development of higher-order cognitive processes, such as executive functioning [12, 13]. However, the associations between signaled night awakening and socio-emotional development in infancy have received little attention even though the bases of social behavior, that is, perception and understanding of social information start to develop during the first year [14]. To address this gap in the literature, we sought to investigate the connections between signaled night awakening and social cognition as well as parent-reported socio-emotional behavior during the first 2 years of life.

Social cognition refers to cognitive processes involved in the perception, processing, interpretation, and storing of information of other people [15]. Accurate processing of social information allows us to recognize the identity of other people and understand their intentions, emotions, and behaviors [16]. Individual differences in social information processing may be related to the way we behave in social situations, for example, through attentional or interpretative biases in processing emotional cues. Already newborns show a visual preference for faces and are sensitive to eye contact [17] and infant-directed speech [18]. Detecting and coordinating eye contact serves as a basis for engaging in social communication, and such ability for joint engagement emerges during the first year of life [19, 20]. In addition, infants’ sensitivity to emotional expressions increases during the second half of the first year [14] and by the end of the first year, infants can use emotional cues to guide their behavior [21–23].

The ability to accurately discriminate between facial expressions is an important aspect of social cognition and it is associated with social adjustment [24]. Experimental studies conducted with adults have suggested that sleep deprivation disturbs the processing of visual emotional information [25, 26] and impairs the recognition of facial expressions [27]. Moreover, a study conducted with adolescents showed that the number of night awakenings and poor sleep quality were negatively associated with the recognition of facial emotions [28]. Less is known, however, about whether difficulties in sleep are associated with social information processing in early childhood, at a time when sleep goes through a wide array of developmental changes.

The number of studies on the connection between sleep and social information processing in childhood is limited, and even less is known about the association between signaled night awakening and social information processing in infancy, even though night awakenings are an age-typical feature of infant sleep [1, 6, 10]. Cremone et al. [29] found that four-year-old nap-deprived children showed a greater attentional bias toward emotional stimuli compared to an after-nap condition. In another study, infants’ better sleep quality (i.e. less waking after sleep onset) and lower sleep-wake pattern variability were connected to infants’ processing of faces and emotional information at the age of 12 months [30]. Infants with higher sleep quality fixated more on the eye region than infants with lower sleep quality, and infants with shorter sleep duration exhibited less autonomic arousal, measured with pupillary reactivity when viewing emotionally negative faces than infants with longer sleep duration. According to these few studies, certain sleep parameters and social information processing seem to be associated in childhood. However, there are hardly any studies on the association between signaled night awakening and social information processing in infancy, during a period of rapid development of social cognition.

In addition to face and emotion processing, sleep disruptions have also been shown to be associated with socio-emotional behavior in adolescents [31, 32], school-aged children [33], and toddlers [6, 34–37]. However, only a few studies have concentrated on signaled night awakening and its connection with socio-emotional behavior in infancy and early childhood. Hysing et al. [38] found that parent-reported night awakening was related to a higher amount of parent-reported overall social-emotional problems at 24 months of age. A recent study from the CHILD-SLEEP birth cohort [39] showed that parent-reported night awakenings at 3, 8, 18, and 24 months of age were associated with greater internalizing and dysregulation symptoms at 24 months of age, whereas night awakenings at 3 and 24 months of age were associated with greater externalizing symptoms. Other studies have found similar associations between night awakenings and socio-emotional problems across the first 5 years [37, 40, 41]. Mindell et al. [34], however, did not find associations between parent-reported night awakenings at 6, 12, and 18 months and parent-reported socio-emotional development at 12 or 18 months of age. Existing studies have also mostly focused on socio-emotional problems, such as externalizing and internalizing, whereas the positive aspects of socio-emotional development, such as social competence, have received less attention.

In the present study, signaled night awakening at 8 months and its associations with social information processing and parent-reported socio-emotional behavior were investigated across the first 2 years of life in two groups differing in the number of night awakenings within the CHILD-SLEEP birth cohort [42]. Social information processing was investigated by using the Overlap task [43, 44], which is a widely used method to study attention to faces and facial emotions in infancy [43, 45, 46]. Previous studies using this paradigm have shown that infants are slower to disengage their attention from faces than non-faces and from fearful as compared to happy, neutral, or angry faces [43, 47, 48]. The attentional bias toward fearful faces (slower disengagement) is typically observed from 5 to 7 months of age [14, 43, 48, 49], or even earlier [50]. According to the limited number of longitudinal studies, attentional bias toward fear seems to attenuate after the first years of life [44, 47]. Importantly, the interindividual variation in attention to faces and particularly fearful faces has also been suggested to be related to different aspects of children’s social development, with a relatively larger bias for fear being associated with secure as compared to insecure attachment [51] and increased altruistic behavior [52] and greater attention to faces being associated with more prosocial behavior [44].

This study had three specific aims. First, we examined whether signaled night awakening is associated with attention to faces longitudinally at 8 and 24 months of age. Second, we examined whether infants with and without signaled night awakenings differed in socio-emotional behavior at 24 months of age (internalizing, externalizing, dysregulation, and social competence). Third, we were interested in whether attention to faces would be associated with the parent-reported domains of socio-emotional behavior. Regarding the hypotheses on the associations between signaled night awakening and attention to faces, we adopted an exploratory approach, since such associations have not been studied previously. We hypothesized, according to previous studies [37–41], that infants with frequent signaled night awakening would exhibit more parent-rated socio-emotional problems at 24 months of age than infants without signaled night awakenings. For the social competence domain, we made no definite hypothesis since previous studies are limited in number and there are hardly any studies, except for Mindell et al. [34], focusing on signaled night awakening and social competence. Finally, regarding associations between attention to faces and parent-reported socio-emotional behavior, prior studies have indicated that robust attention biases to faces and negative emotions are normative during early development, and the strength of such biases may even be associated with positive outcomes [44, 51, 52]. On the other hand, a large body of literature has demonstrated associations between attention to negative emotions and internalizing and anxiety problems in adults and older children [53]. Consequently, we hypothesized that greater attention to faces would be associated with better social competence, but made no definite hypothesis concerning the emotional valence of faces, or whether attention to faces would be associated with negative aspects of socio-emotional behavior.

## Methods

### Participants

The current sample of infants was recruited within the CHILD-SLEEP birth cohort (*n* = 1667) in which different sleep characteristics and their associations with multiple aspects of child development are being studied [42, 54]. The participating families filled questionnaires before the birth of their infants and when the infants were 3, 8, 18, and 24 months old [42]. Parents of a subsample of the cohort (*n* = 406) were contacted when the child was 8 months through phone calls and asked how many times their infant had typically woken up during the night (24.00–06.00) and needed soothing during the past 2 weeks. The inclusion criteria for the current sample was that the 8-month-old infants had three or more signaled night awakenings or no more than one signaled night awakening. The infants with three or more signaled night awakenings formed the waking group. Infants with no more than one signaled night awakening formed the nonwaking group [12, 54]. The exclusion criteria were prematurity or native language other than Finnish. In addition, infants with two signaled night awakenings were excluded from the study to have two distinct groups. The Ethical Committee of the Pirkanmaa Hospital District (ETL-code: #R11032) approved the study protocol, and all participating families signed a written consent form before participation. In total, 146 White Finnish infants participated at 8 months of age, with 77 infants in the waking group (mean age = 8.5 months, *SD* = 0.4 months) and 69 infants in the nonwaking group (mean age = 8.6 months, *SD* = 0.4 months). Two additional participants were examined but were excluded from further analysis due to prematurity (*n* = 1) and the parents’ native language being other than Finnish (*n* = 1).

The demographic statistics of the sample are shown in Table 1. At 8 months of age, the two groups differed in the amount of breastfeeding (*X*^2^ (1, *n* = 133) = 13.413, *p* = .001), co-sleeping (*X*^2^ (1, *n* = 119) = 20.376, *p* < .001), and the infant’s ability to fall asleep alone (*X*^2^ (1, *n* = 133) = 16.454, *p* < .001) [12]. The infants in the waking group were more likely to be breastfed, to co-sleep with their parents, and less able to fall asleep alone than the infants in the nonwaking group. Breastfeeding had three categories: the infant was only breastfed, breastfed and formula fed, or only formula fed. Co-sleeping was dichotomized as co-sleeping with the parent less than twice in a month versus at least once every week. The ability to fall asleep alone was dichotomized as the infant was able to fall asleep alone only once a week versus at least once a day. These three factors were included in the analysis as covariates to statistically control for potential confounding effects. Participants of the current study were randomly recruited from the prevention sub-study of the main cohort in which part of the families received psychoeducation through brochures on how to support infant sleep quality. In the control group, the families participated in standard well-child visits. The infants in the waking group and nonwaking group came from both the prevention and control healthcare centers (*X*^2^ (1, *n* = 146) = 3.394, *p* = .065). The healthcare center status was included as a covariate in our analyses to control for its potential effects on the results. Additional parent-reported and actigraphy-based sleep duration and quality parameters are shown in Table 2 according to the group. Our previous work showed that based on parent reports, the infants in the waking group slept less in total and they spent more time awake during the night than the nonwaking group [54], but they did not differ in actigraphy-based sleep duration measures [12]. In addition, the waking group had more parent-reported signaled night awakenings than the nonwaking group at 18 and 24 months of age [54]. The group formation at 8 months was also validated with actigraphy-based night awakening data which showed that the waking group had more night awakenings than the nonwaking group when measured with actigraphy [12].

**Table 1.** Descriptive statistics for the two groups according to questionnaire data

	Waking group		Nonwaking group		
	*n*	%	*n*	%	*P*
Gender					.889
Boys	43	56.6	35	50.7	
Girls Missing 6	33	43.4	34	49.3	
Siblings					.354
Yes	27	36.5	33	34.2	
No Missing 10	47	63.5	34	50.7	
Mother’s education					.210
>15 years	36	47.4	31	45.2	
<15 years Missing 7	40	52.6	37	54.8	
Father’s education					.671
>15 years	55	70.5	45	70.3	
<15 years Missing 12	23	29.5	16	25.0	
Mother’s monthly net income					.377
>2000 €	23	29.5	23	35.9	
1000–2000 €	39	50.0	24	37.5	
<1000 € Missing 14	14	17.9	14	21.9	
Father’s monthly net income					.492
>2000 €	49	62.8	42	65.6	
1000–2000 €	20	25.6	16	25.0	
<1000 € Missing 13	8	10.3	3	4.7	
Breastfeeding					.002
Breastfed only	43	58.9	20	33.3	
Breastfed and formula fed	16	21.9	11	55.0	
Formula fed only Missing 18	14	19.2	29	48.3	
Co-sleeping					<.001
>once in a week	29	44.6	4	7.4	
<2 times a month Missing 32	36	55.4	50	92.6	
Falling asleep alone					<.001
Once in a week	49	68.1	20	32.8	
Once in a day Missing 18	23	31.9	41	67.2	
Health care center					.065
Prevention	43	55.8	28	40.6	
Control Missing 5	34	44.2	41	59.4	

**Table 2.** Descriptive statistics of parent-reported sleep and actigraphy-based sleep parameters at 8 and 24 months of age

	Waking group			Nonwaking group		
	*n*	*M*	*SD*	*n*	*M*	*SD*
**8 months**						
*Parent-reported*						
Duration of nocturnal sleep (min)	75	589.9	58.4	60	597.8	55.7
Duration of daytime sleep (min)	76	188.4	53.6	60	204.8	63.1
Duration of total sleep (min)	75	778.8	70.9	60	802.6	60.5
Sleep latency (min)	74	24.8	16.9	57	18.2	15.5
Time spent awake at night (min)	69	30.6	25.7	48	15.8	20.6
Night awakenings (count)	73	3.8	1.9	54	0.9	0.6
Proportion of nighttime sleep (%)	75	75.9	5.8	60	74.7	6.6
*Actigraphy-based*						
Actual sleep time (min)	67	511.1	55.0	61	518.5	56.6
Sleep latency (min)	66	19.3	19.3	58	17.8	22.1
Sleep efficiency (%)	66	78.7	6.2	58	79.1	6.8
Proportion of nighttime sleep (%)	37	85.7	5.7	31	85.1	6.6
**24 months**						
Duration of nocturnal sleep (min)	53	589.4	46.4	41	605.5	37.9
Duration of daytime sleep (min)	53	105.4	38.8	41	114.6	46.1
Duration of total sleep (min)	53	694.8	45.3	41	720.6	55.5
Sleep latency (min)	48	23.9	18.1	38	17.9	12.5
Time spent awake at night (min)	46	11.8	12.3	38	4.0	9.0
Night awakenings (count)	50	1.0	0.9	40	0.6	0.6
Proportion of nighttime sleep (%)	53	84.9	5.1	41	84.3	5.1
*Actigraphy-based*						
Actual sleep time (min)	35	539.7	43.0	28	542.8	46.6
Sleep latency (min)	36	26.6	18.7	28	22.3	15.0
Sleep efficiency (%)	36	81.7	5.6	28	83.8	4.2
Proportion of nighttime sleep (%)	31	87.4	7.4	17	89.4	5.9

At 24 months of age, 65 infants from the waking group (mean age = 24.6 months, *SD* = 2.2 months) and 56 infants from the nonwaking group (mean age = 24.5 months, *SD* = 2.9 months) returned for another assessment (*n* = 121, 83% retention rate). Attrition was not related to the performance in the Overlap task at 8 months, all *p*s > .411.

### Procedure

The current sub-sample of infants participated in assessments at 8 and 24 months of age. At both assessment points, there were two separate research visits. During the first research visit, psychomotor development was measured [54]. On the second research visit, executive functioning [12], parent-infant interaction, and heart-rate variability (both of which are not reported here), and attention to emotional faces were measured. Socio-emotional behavior was measured with a parent rating when the children were 24 months old as a part of a larger online questionnaire sent to the whole CHILD-SLEEP cohort.

The second research visit was conducted in the laboratory. The child sat on the parent’s lap approximately 60-cm from the computer screen. The computer screen was a 19-inch screen surrounded by a black frame. At 8 months of age, a video camera was hidden on top of the computer screen for the experimenter to observe the child. In addition, the video recordings of the Overlap task were saved for offline eye movement analyses. When the children were 24 months old, the stimuli were presented from a 21-inch screen with a video camera on top of it. Eye-tracking measurement was added to the protocol at 24 months and attention to faces was measured using a corneal reflection eye-tracker (Tobii TX300; Tobii Technology, Stockholm, Sweden). Stimulus presentation was controlled at both ages with E-prime 2.0 software (Psychology Software Tools, Pittsburgh, PA). Before starting the experiment at 24 months, the eye-tracker camera was calibrated with the Tobii five-point calibration procedure in which a cat appeared with a beep sound to every corner and to the center of the screen. In the case of unsatisfactory calibration data for the five locations (i.e. one or more calibrations were missing or were not properly calibrated), the calibration was repeated a maximum of two times. After these attempts, if one or more calibrations were still missing, the last attempt was accepted, and the presentation of the task was initiated. Children were not excluded from the sample based on the lack of calibration points because the analysis of the primary outcome of the task (i.e. gaze disengagement and shift between two objects that were separated in space) was not reliant on precise spatial tracking accuracy and has been found to be robust against variations in calibration quality [55].

Before starting the task, the lights were dimmed. At both ages, parents were instructed not to interact with their child unless it was necessary to soothe the child during the presentation of tasks. Each trial in the Overlap task started with a red circle appearing on a gray background. The red circle expanded from 0.4° to 4.3° in a continuous fashion for the child to focus their eyes on the screen. When the child’s eyes were focused on the screen, the experimenter initiated the following trial. If the child did not focus on the screen, the experimenter called the child by name and initiated the trial only after the child focused on the screen.

### Measurements

#### Attention to emotional faces

**Figure 1.**
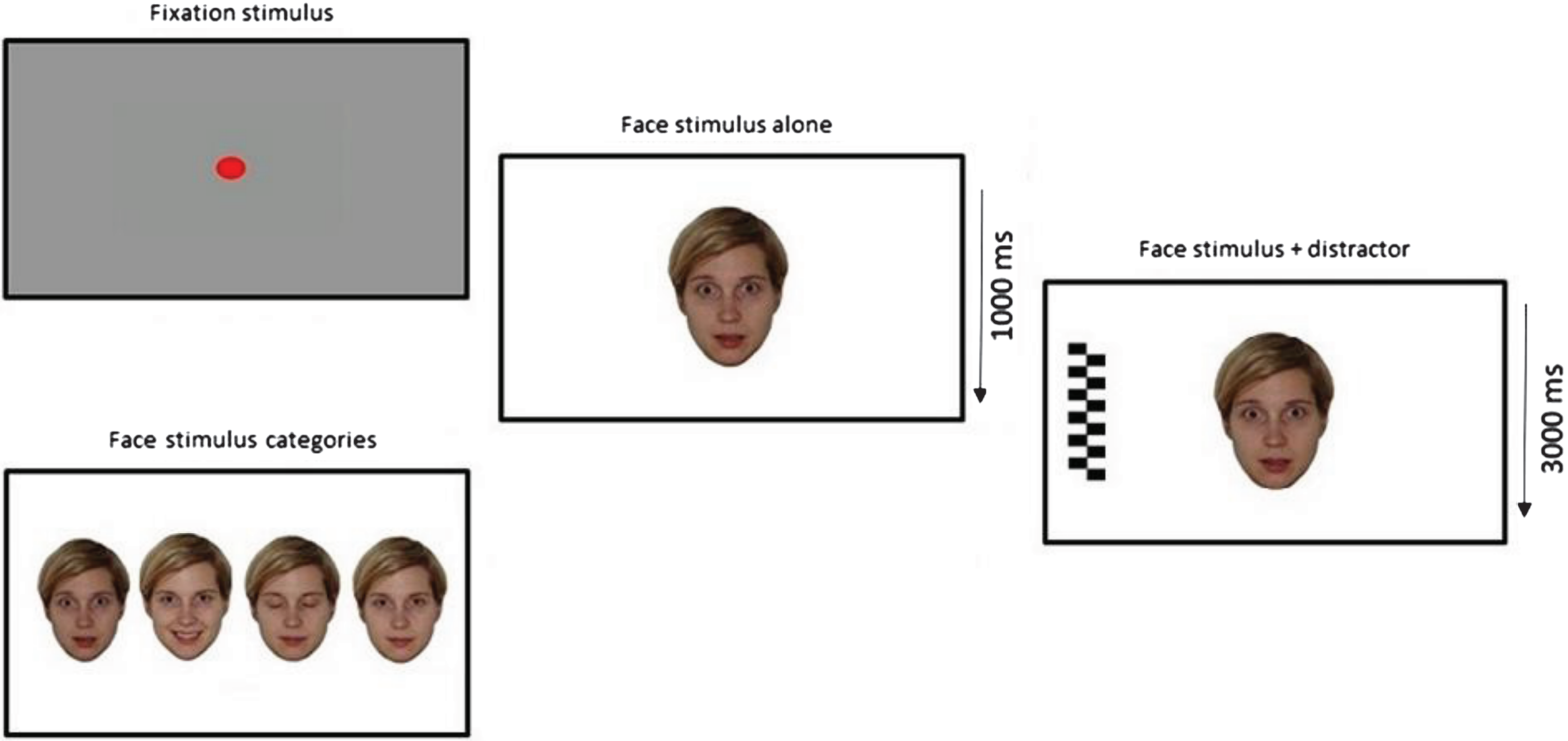
An example of a trial in the overlap task and the different face stimulus categories used.

Attention to emotional faces was measured with the Overlap task. The face stimuli consisted of four different color images of one female model. The model depicted happy, fearful, and neutral with eyes open and neutral with eyes closed expressions (Figure 1). In addition, two different distractor stimuli (black-and-white checkerboard and a circle pattern) were used. After the initiation of a trial in the Overlap task, the facial stimulus (15.8° × 11.4°) appeared on the center of the screen for 1000 ms on a white background. Next, the facial stimulus was flanked by a lateral distractor stimulus (15.8° × 4.0°) that appeared either to the left or to the right side of the screen. The facial stimulus and the distractor were visible for 3000 ms. The distance between the face and distractor stimulus was 13.6°. The presentation order of the stimuli was randomized with the restrictions that the same emotional face was repeated no more than four times in a row, and the distractor appeared at the same location no more than three times in a row. Altogether, there were 24 trials, that is, 6 for each of the 4 different face conditions. At 24 months, the distractor stimulus was modified to have colors to make it more interesting to the child with two different color schemes (blue–red and green–yellow). In addition, these color schemes alternated rapidly, giving the impression that the distractor stimulus was animated. An example of a trial in the Overlap task is shown in Figure 1.

#### Socio-emotional behavior

Socio-emotional behavior was measured using the Brief Infant-Toddler Social and Emotional Assessment (BITSEA) questionnaire at 24 months of age [56]. A Finnish translation of the questionnaire [57] was used with the original US norms. The BITSEA consists of 42 statements, with 31 items measuring socio-emotional problems and 11 items measuring socio-emotional competence. Parents answer with a three-point scale (not true/rarely = 0, somewhat true/sometimes = 1, very true/always = 2). Externalizing, internalizing, dysregulation, and social competence domains were derived from the questionnaire. Internal consistency scores showed moderate internal consistency (Externalizing, α = .650, Internalizing, α = .515, Dysregulation, α = .660, and Social Competence, α = .657). These values are consistent with the original US norms for Social Competence [58] and somewhat lower than the alphas reported for the Internalizing and Externalizing domains [59]. Altogether, 53 participants from the waking group and 47 participants from the nonwaking group provided data for the BITSEA questionnaire.

### Data analysis

#### Video coding at 8 months.

Eye movements in the Overlap task were analyzed using VirtualDubMod software 1.5.10.2 (http://virtualdubmod.sourceforge.net/). A trained observer who was unaware of the child’s group status coded all the videos offline. Eye movements were examined manually, frame by frame, with one frame lasting 40 ms. The procedures of previous studies using the same methodology [43, 60] were followed. If the child did not focus on the screen at the beginning of the trial, the trial was rejected. The child’s saccades during a trial were coded to calculate dwell times for different face conditions. The duration of attention dwell time on the stimulus (happy, fearful, neutral with eyes open, and neutral with eyes closed) was determined for the period starting 160 ms from the onset of the lateral distractor stimulus and ending 1000 ms after distractor stimulus onset. Following previous studies [55], the duration was then converted to a normalized dwell time index score using the following formula:


$$\begin{array}{l} Dwelltimeindex=\frac{\overset{n}{\underset{i=1}{\sum }}\left(1-\frac{1000-x_{i}}{840}\right)}{n} \end{array}$$

In the formula, *x* is the time point of saccadic eye movement (i.e. the last time point when the gaze is in the area of the first central stimulus preceding a saccade toward the distractor stimulus) and *n* is the number of scorable trials in a given stimulus condition. In this index, the shortest acceptable saccadic eye movement latency (160 ms) results in a score of 0, and the longest possible latency (or a lack of saccade, which is equal to the last measured time point of the first stimulus at 1000 ms) with a score of 1. The dwell time indices were calculated separately for each of the four stimulus conditions (i.e. happy, fearful, neutral with eyes open, and neutral with eyes closed). Trials with anticipatory eye movements (<160 ms), excessive movements during the trial, and saccades not directed toward the distractor stimulus were rejected. At least two scorable responses per face condition were required to be included in the analyses. At 8 months, the average number of scorable trials in the different face conditions were: happy: 5.4 (waking group) and 5.3 (nonwaking group); fear: 5.5 (waking group) and 5.3 (nonwaking group); neutral with eyes open: 5.4 (waking group) and 5.2 (nonwaking group); and neutral with eyes closed: 5.4 (waking group) and 5.2 (nonwaking group). At 8 months of age, 74 participants in the waking group and 62 participants in the nonwaking group provided at least two scorable trials in each face condition and were included in the analysis.

#### Eye movement coding at 24 months

. The eye-tracking data of the Overlap task at 24 months of age were saved as text files and analyzed using a library of MATLAB routines for offline analysis of raw gaze data [55]. These files included timestamps corresponding to the onset times of the central and lateral stimuli. Data analysis of the saccadic eye movements from the central stimulus to the distractor was implemented by automatic coding of the x and y coordinates of the eye-tracking data. Trials with a sufficient length of fixation on the central stimulus (i.e. > 70% of the time) during the time preceding gaze disengagement or the 1000-ms timeout, sufficient number of valid samples in the gaze data (i.e. no gaps > 200 ms), and valid information about eye movement from the central to the lateral stimulus (i.e. the eye movement did not occur during a period of missing gaze data) were retained for analysis. The dwell times were then calculated in the same way as in the 8-month coding, separately for each face condition. The only difference was the threshold of anticipatory eye movements, which was set at 150 ms in the 24-month data. In previous research, automated and manual coding of attention disengagement has shown high (over 97%) agreement [55]. At 24 months, the average number of scorable trials in the different face conditions were: happy: 4.5 (waking group) and 4.6 (nonwaking group); fear: 4.8 (waking group) and 4.5 (nonwaking group); neutral with eyes open: 4.5 (waking group) and 4.5 (nonwaking group); and neutral with eyes closed: 4.4 (waking group) and 4.3 (nonwaking group). Successful data at 24 months of age were gathered from 64 participants in the waking group and 55 participants in the nonwaking group.

### Statistical analysis

Statistical analyses were performed using SPSS version 25 (IBM Corp., Armonk, NY). All outcome variables were continuous. The distribution of the variables was visually screened using boxplots and potential outliers were identified using scatterplots. No extreme values were observed. In addition, in the Overlap task, dwell times were also converted to *z*-scores and no outliers were observed, as all the values were within *z* = ±3.29 [61]. To analyze the Overlap data longitudinally, a Linear Mixed Model (LMM) was used to maximally utilize the incomplete longitudinal data. As fixed factors, both within-subject and between-subject effects and their interactions were analyzed. Age (8 and 24 months) and emotion (happy, fearful, neutral with eyes open, and neutral with eyes closed) were used as within-factors. The between-factor group consisted of the waking and nonwaking groups. In addition, their interactions were included in the model. In the case of statistically significant main effects, Bonferroni-corrected post hoc tests were used. Cohen’s *d* was used as an effect size indicator for the pairwise comparisons. First, a model with all the covariates such as breastfeeding, co-sleeping, ability to fall asleep alone, healthcare center status, and sum scores of questionnaires assessing maternal depressive (CES-D) [62] and anxiety (STAI) [63] symptoms at 8 months and within- and between-factors was performed. None of the covariates used were statistically significant; therefore, they were not included in the final model. Akaike’s information criterion was used in the model and covariance structure selection. The group differences in the BITSEA subdomains were analyzed using *t*-tests for independent samples. Finally, the connections between dwell times in the different face conditions in the Overlap task at 8 and 24 months and the BITSEA subdomains at 24 months were analyzed with Pearson correlations. The alpha level for statistical significance was set at *p* < .05.

## Results

**Figure 2.**
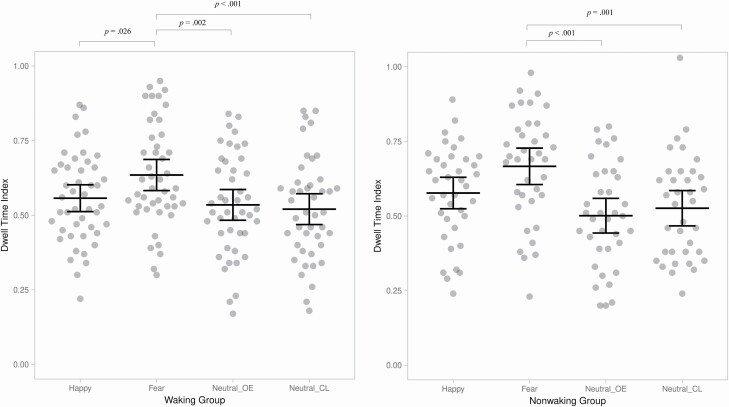
Individual dwell times and their means (and 95% CI) to different faces according to group (The data were visualized by an R Shiny app by Postma and Goedhart [64].).Neutral_OE, neutral with open eyes; Neutral_CL, neutral with closed eyes.

The LMM of the longitudinal Overlap data revealed a main effect of emotion, *F*(3, 114.319) = 21.544, *p* < .001. Dwell times were longest in the fearful face condition (*M* = .629, SE = .017) as compared to the happy face (*M* = .576, SE = .015), *p* = .005, *d* = .33, neutral face with eyes open (*M* = .525, SE = .017), *p* < .001, *d* = .62, and neutral face with eyes closed (*M* = .527, SE = .016), *p* < .001, *d* = .62, conditions. In addition, dwell times were longer in the happy face condition than in the neutral face with eyes open, *p* < .001, *d* = .32, and in the neutral face with eyes closed, *p* = .008, *d* = .32, conditions. A group × emotion interaction was also observed, *F*(3, 114.319) = 3.615, *p* = .015. Bonferroni-corrected pairwise comparisons indicated that the waking and nonwaking groups had a different pattern of dwell times across the four face conditions (see Figure 2). In the waking group, dwell times were longer in the fearful face condition (*M* = .611, SE = .024) than in the happy face (*M* = .550, SE = .020), *p* = .026, *d* = .45, neutral face with eyes open (*M* = .541, SE = .022), *p* = .002, *d* = .49, and neutral face with eyes closed (*M* = .505, SE = .022), *p* < .001, *d* = .71, conditions. No other differences were statistically significant, all *p*s > .145. In the nonwaking group, however, dwell times in the fearful face condition (*M* = .646, SE = .025) differed from the neutral face with eyes open (*M* = .510, SE = .024), *p* < .001, *d* = .81, and neutral face with eyes closed (*M* = .551, SE = .023), *p* = .001, *d* = .58, conditions, but not from the happy face condition (*M* = .602, SE = .022), *p* = .315 *d* = .28. In addition, dwell times in the happy face condition differed from the neutral face with eyes open condition, *p* < .001, *d* = .58, in the nonwaking group. In other pairwise comparisons, the differences were not statistically significant, all *p*s > .109. The main effect of group, *F*(1, 113.633) = .868, *p* = .354, and the group × age, *F*(1, 313.943) = .028, *p* = .866, and group × age × emotion, *F*(3, 203.264) = .458, *p* = .712, interactions were not significant and therefore the interactions were not included in the final model.

Parent-reported socio-emotional behavior was analyzed separately for the different subdomains, and the descriptive statistics for both groups are presented in Table 3. The two groups differed in the dysregulation domain, *t*(97)= 3.542, *p* = .002, *d* = .72 and social competence domain, *t*(96) = −1.998, *p* = .049, *d* = .48, with the waking group having more dysregulation problems and lower social competence scores than the nonwaking group. In the externalizing domain, the two groups did not differ, *t*(96)= .930, *p* = .355. The difference between the waking and nonwaking groups was, likewise, not statistically significant in the internalizing domain *t*(94.992)= 1.874, *p* = .065, *d* = .38, even though the waking group appeared to have slightly more internalizing problems than the nonwaking group. The dysregulation subdomain of the BITSEA contains two items concerning sleep “Wakes up at night and needs help to fall asleep again” and “Has trouble falling asleep or staying asleep.” Since the waking and nonwaking groups differed in the number of signaled night awakenings, the analysis of the dysregulation domain was conducted again with the two items concerning sleep removed to ensure that the differences in the dysregulation domain were not merely a reflection of night awakening. The differences between the two groups in the dysregulation domain remained even after the removal of the two sleep items, even though the difference between groups was smaller, *t*(97) = 1.991, *p* = .049, *d* = .40.

**Table 3.** Means and standard deviations of domains of socio-emotional behavior in the BITSEA questionnaire for the two groups

BITSEA domain	Waking group		Nonwaking group		
	*M*	*SD*	*M*	*SD*	*P*
Dysregulation	4.23	2.74	2.47	2.14	.002
Social competence	18.11	2.42	19.13	2.60	.049
Externalizing	3.41	2.27	3.00	2.10	.355
Internalizing	1.59	1.75	1.05	.99	.065
Dysregulation (no sleep)	2.42	1.81	1.79	1.53	.049

The correlational analyses between dwell time and BITSEA subdomains showed that dwell times in the happy face condition, but not other face conditions, correlated with different BITSEA subscales (Table 4). More specifically, dwell times in the happy face condition at 8 months of age correlated positively with the social competence domain, *r*(62) = .337, *p* = .007, and with the externalizing domain, *r*(62) = .260, *p* = .041. Dwell times in the happy face condition at 24 months of age correlated negatively with the dysregulation scores (with the two sleep items removed), *r*(85) = −.214, *p* = .049.

**Table 4.** Correlations between attention dwell times to faces at 8 and 24 months and BITSEA domains at 24 months of age

	Internalizing	Externalizing	Dysregulation (no sleep)	Social competence
**8 months**				
Happy face	.203	.260*	−.031	.337**
Fearful face	.242	.157	−.096	.047
Neutral with eyes open	.150	.174	.019	.072
Neutral with closed eyes	.146	.155	.050	.228
**24 months**				
Happy face	−.135	.204	−.214*	.132
Fearful face	−.096	.042	−.096	.130
Neutral with eyes open	−.088	.131	−.092	.069
Neutral with closed eyes	−.070	−.037	−.087	.047

*Correlation is significant at the .05 level.

**Correlation is significant at the .01 level.

The correlations are unadjusted for multiple comparisons.

## Discussion

In the present study, signaled night awakening and its associations with social information processing and socio-emotional behavior were investigated across the first 2 years of life. Children were divided into two groups based on the number of signaled night awakenings reported by their parents at 8 months of age. In a longitudinal design, social information processing was measured at 8 and 24 months of age with a task measuring attention to emotional and neutral faces. In addition, socio-emotional behavior was assessed with a parent-rated questionnaire at 24 months of age. Regarding links between signaled night awakening and attention to faces, we adopted an exploratory approach, since such associations have not been studied previously. In addition, based on previous studies, we hypothesized that signaled night awakening would be associated with more parent-rated socio-emotional difficulties [37–41]. For the hypothesis regarding associations between attention to faces and socio-emotional behavior, we hypothesized that attention to faces would be associated with better social competence, as suggested by limited prior evidence [44, 51, 52], but made no definite hypothesis concerning the emotional valence of faces, or whether attention to faces would also be associated with negative aspects of socio-emotional behavior.

Regarding social information processing, our results replicated the robust finding of longer attention dwell times toward fearful faces as compared to happy or neutral faces [44, 48, 51, 60]. The attentional bias toward fearful faces during the second half of the first year has been indicated as a marker of normative social information processing in infancy and has been suggested to be associated with positive aspects of social development, such as attachment security and prosociality [44, 51, 52].

Importantly, our study also sheds new light on the associations between signaled night awakening and social information processing. In the waking group, dwell times to fearful faces were longer than dwell times to happy or both types of neutral faces whereas in the nonwaking group, dwell times to fearful faces were longer than dwell times to neutral faces, but dwell times between fearful and happy faces did not differ. Thus, in the waking group, the attentional bias to fearful faces in relation to other face types was more pronounced than in the nonwaking group. A previous study showed that naps reduced attention bias to emotional stimuli in 4-year-old children [29]. Our results add to previous work by showing that signaled night awakening may alter attention to emotional stimuli in very young children, with children reported to have fewer signaled night awakenings showing a less pronounced attention bias to fearful faces.

As previously mentioned, attentional bias toward fearful faces may be a normative aspect of early social information processing [43, 44, 48]. This pattern of attention was evident in the waking group, whereas in the nonwaking group, dwell times to fearful and happy faces did not differ. To understand this finding, it is important to note that our analysis of the Overlap task was longitudinal, with the attention patterns observed in data averaged across 8 and 24 months of age because no interactions involving age were observed. Given that longitudinal studies suggest that the robust attention bias to fearful faces observed in infancy may become smaller and less selective to fearful faces across early childhood [44, 47, 65], it would be intriguing to speculate whether the group differences observed in the present study reflect differential age-related patterns of attention bias. However, this possibility remains to be determined, as the interaction between group, emotion, and age was not significant in the present analysis. The longitudinal effects of signaled night awakening on social information processing remain an important topic for future studies.

As hypothesized, signaled night awakening was connected to different aspects of parent-reported socio-emotional behavior at 24 months of age. The two groups differed in the amount of dysregulation, with the waking group having more problems than the nonwaking group. This finding is consistent with the results of Morales-Muñoz et al. [39] from the current cohort, showing that night awakenings at 3, 8, 18, and 24 months of age were related to greater dysregulation [39], but inconsistent with those of Mindell et al. [34], according to which night awakening at 6, 12, and 18 months was not associated with dysregulation at 12 or 18 months of age. In the BITSEA questionnaire the dysregulation scale consists of items concerning regulation difficulties in sleep, eating, and emotional or self-regulation difficulties (“Cries or tantrums until s/he is exhausted,” “Has trouble adjusting to changes,” and “Often gets very upset”). In previous literature, frequent signaled night awakenings have been thought to be associated with the inability to soothe or regulate oneself back to sleep after waking. Thus, the ability to fall asleep alone is a highly important developmental task, for which self-regulation skills are essential. Our results extend previous work by showing that children with frequent signaled night awakenings at 8 months have persistent difficulties in regulating their behavior more generally and not only in terms of regulating sleep since the two groups differed in dysregulation even after the removal of the two items concerning sleep difficulties and night awakening. In future studies, longitudinal associations with signaled night awakening and different aspects of self-regulation should be further investigated to determine whether such early difficulties in behavioral regulation are precursors for later, more severe problems of self-regulation.

Contrary to our expectation, the waking and nonwaking groups did not differ in the amount of externalizing problems at 24 months of age, and the difference in internalizing problems was only marginal. Our results are consistent with a previous study with an equivalent sample size showing that night awakening was not associated with internalizing or externalizing problems [34], but discrepant with large community-based studies, including the full CHILD-SLEEP cohort, which showed night awakening to be connected to both externalizing and internalizing difficulties [37, 39, 41]. In these larger studies, stronger associations have been found for internalizing compared to externalizing difficulties. It could be that the effect of signaled night awakening on internalizing and externalizing problems during early development is relatively small and possibly confounded by other factors. Therefore, such associations may be more difficult to detect in studies with smaller sample sizes. Furthermore, it is also possible that the associations between signaled night awakening and internalizing and externalizing difficulties become stronger with age.

Interestingly, in our study, the waking group had lower parent-reported social competence than the nonwaking group. Our finding is consistent with a recent study showing that night awakening is associated with lower social competence [34]. In that study, the frequency of night awakening and the longest stretch of sleep, but not total sleep duration, bedtime, or sleep onset latency, were the only sleep parameters associated with social competence at the concurrent age of 12 months. Results from other studies reporting associations between sleep duration and social competence in children have been mixed [66–68]. Early childhood is an important period for the development of social competence. According to our study and that by Mindell et al. [34], night awakening may be associated with slower development of social competence already during the first 2 years. Prior studies have mainly focused on the associations between sleep and socio-emotional problems, whereas studies focusing on the positive aspects of socio-emotional development are limited in number. In future studies, it is important to recognize possible socio-emotional problems as early as possible, but also to extend the focus to the positive aspects of socio-emotional development. In our longitudinal cohort, we can follow these children through childhood to find out whether these differences in social competence will even out or become larger with age. In addition, the neurocognitive mechanisms by which night awakenings influence social competence are an important target for future studies, and the possible mediating role of behavioral regulation difficulties in the development of social competence should be considered.

Taken together, the findings of this study suggest that children with frequent signaled night awakenings at 8 months are at increased risk for behavioral regulation problems and the development of poorer social competence. In addition, attention to emotional faces, but not attention to faces in general, was associated with signaled night awakening. In our previous work, the same children with frequent signaled night awakenings also exhibited lower executive functioning performance than children without signaled night awakenings [12], but they did not show differences in overall psychomotor development [54]. Therefore, emotional and executive functioning may be especially associated with signaled night awakening in infancy, and children who signal their awakenings may have difficulties in self-regulation in multiple developmental domains. Previous studies have shown that signaled night awakening can be successfully diminished or treated with interventions that focus on parent training strategies [69]. Whether changes in signaled night awakening following intervention also produce changes in social information processing and socio-emotional behavior would be an important question to understand the mechanisms linking signaled night awakening to social behavior.

Regarding the last research question, our findings suggested that attention to faces and parent-rated socio-emotional behavior may be associated. Attention toward happy faces at 8 months correlated positively with increased social competence and greater externalizing problems at 24 months of age. Concurrently at 24 months of age, greater attention toward happy faces was associated with lower scores on the dysregulation domain. These findings are partially consistent with those of Peltola et al. [44], which indicated that infants’ greater attention to faces is associated with prosocial behavior. Attentional biases toward positive stimuli have been observed in typically developing children [70] and individual differences in attentional bias to positive information have been associated with stronger positive affective responses [71] and increased prosocial behavior, less externalizing disorders, and less emotionally withdrawn behavior in foster care children [72]. Our results may thus suggest that greater attention toward positive stimuli is associated with better socio-emotional outcomes, which may reflect the child’s interest in the rewarding features of interactions. Surprisingly, our results also showed that greater attention to happy faces was associated with greater externalizing behavior. However, it is important to keep in mind that, in our sample, the children were 2 years old and the overall scores on the externalizing domain were low, and therefore it might be that the associations with the externalizing domain could be driven more by moderately elevated impulsive behavior than acting-out or aggressive behavior, which are descriptive of high externalizing scores in early childhood. A limitation of the current correlational results is that the correlations were not adjusted for multiple comparisons. In addition, the sample size in these correlation analyses was somewhat underpowered to detect small (i.e. *r* < .30) correlations. Therefore, these exploratory analyses should be interpreted with caution. It should also be noted that our findings concern children under 2 years of age and in future studies it will be important to examine whether similar findings are observed in older children and more versatile and larger samples.

As a limitation, our group formation relied on parent reports. The two groups were formed at 8 months of age since our interest was to study signaled night awakening already in infancy. We also analyzed actigraphy data at 8 months of age [12] and showed that the waking and nonwaking groups differed also in actigraphy-based night awakenings at 8 months, thus validating our group formation based on parent reports. The two groups, however, did not differ in other actigraphy-based sleep duration measures. In addition to 8 months, signaled night awakening was also assessed longitudinally with questionnaires at 18 and 24 months of age, and the waking and nonwaking groups differed in parent-reported signaled night awakening at 18 and even at the age of 24 months, thus the two groups were distinguished by the number of signaled night awakenings also later in infancy. One specific feature of the current study was that the participants were drawn from the prevention substudy of the larger cohort. Thus, half of the participants were from the so-called prevention health care centers and the rest from the control health care centers. The families in the prevention health care centers received preventive psychoeducation on infant sleeping habits through brochures, whereas the control health care centers received no preventive sleep material. Infants in both night awakening groups came from both of these health care centers. In addition, the health care status of the child was taken into account in our analyses, and it did not have a significant effect on the results. In addition, our previous work showed that children’s health care center status was not associated with different sleep quality and duration parameters, psychomotor development [54], or executive functioning [12]. As another potential limitation, we relied on parental reports of socio-emotional behavior. Therefore, it is possible that children with frequent signaled night awakenings are perceived as generally more dysregulated by their parents due to more frequent signaled night awakenings. However, in the present study, this seems unlikely since the differences between groups in dysregulation remained even though the questions concerning night awakening were removed. Future studies would, however, benefit from using multiple informants such as daycare staff in addition to parents, and the development of more objective experimental methods for studying socio-emotional development would also improve the reliability of this line of research. An important factor potentially affecting attention biases in early development is infant temperament (e.g. negative affectivity or behavioral inhibition), which was outside the scope of the present paper and thus not included in the analyses. In the future, it will be important to characterize the associations between temperament and attention biases with sufficiently large samples covering representative variation in relevant temperament dimensions to evaluate whether findings linking temperament and attention biases in older children [73] are replicated in infants. Finally, our sample consisted of White families of mainly middle-class origin and therefore the generalizability of our results to other ethnicities and sociodemographic groups is limited. For example, co-sleeping and signaled night awakening can be considered to reflect normative patterns of sleeping in infancy in other cultures than our sample that represents a Western culture.

In conclusion, our results showed that signaled night awakening is associated with social information processing and socio-emotional behavior already during the first 2 years of life. Children with frequent signaled night awakenings exhibited different patterns of attention to emotional faces than children without signaled night awakenings. In addition, at 24 months of age, children with frequent signaled night awakenings had greater parent-reported regulation difficulties and lower social competence than children without signaled night awakenings. However, no clear differences in internalizing or externalizing problems were found between the two groups. Attention to happy faces at 8 and 24 months of age was connected to parent-rated socio-emotional behavior at 24 months. According to our results, it is possible that signaled night awakening could be an early marker of ineffective self-regulatory abilities that predispose children to later socio-emotional behavioral problems. However, the differences between groups were rather small, and longitudinal studies are needed to determine whether the differences in social information processing and socio-emotional behavior will even out or increase as children grow older. Nevertheless, our study provides a novel contribution regarding the influence of signaled night awakening on children’s social information processing and social development.
